# A Halophilic Bacterium for Bioremediation of Saline–Alkali Land: The Triadic and Synergetic Response Mechanism of *Oceanobacillus picturae* DY09 to Salt Stress

**DOI:** 10.3390/microorganisms13071474

**Published:** 2025-06-25

**Authors:** Tianying Nie, Liuqing Wang, Yilan Liu, Siqi Fu, Jiahui Wang, Kunpeng Cui, Lu Wang

**Affiliations:** Institute of Biomedical Engineering, College of Life Sciences, Qingdao University, Qingdao 266071, China; ying18854709906@163.com (T.N.); 15751537912@163.com (L.W.); liuyilan2022@163.com (Y.L.); fuussq@163.com (S.F.); jhwang@qdu.edu.cn (J.W.); cuikunpeng@qdu.edu.cn (K.C.)

**Keywords:** halophilic bacteria, salt tolerance mechanism, ion steady state, regulation of osmosis

## Abstract

The strain of *Oceanobacillus picturae* DY09, as a typical halophilic microorganism, possesses distinctive salt adaptation mechanisms that hold significant application value in the fields of agriculture, industry, and biomedicine. To deeply analyze the salt-tolerance molecular mechanism of this strain, this research disclosed its salt-tolerance strategies under diverse salt concentrations through transcriptomics. In a low-salt environment, the DY09 strain adopted a “metabolic simplification” strategy, significantly reducing the metabolic load by promoting lysine degradation and inhibiting the biosynthesis of branched-chain amino acids and glycine betaine (GB) but upregulating the expression of the GB transporter gene *betH* and preferentially utilizing exogenous GB to maintain basic osmotic balance. When exposed to high-salt stress, this strain activated multiple regulatory mechanisms: it upregulated the expression of Na^+^/K^+^ antiporter proteins to maintain ionic homeostasis; the synthesis genes of amino acids such as arginine and proline were significantly upregulated, and the GB synthesis genes *betA*/*B* and the transporter gene *betH* were upregulated concurrently, which realized the synergistic operation of endogenous synthesis and exogenous uptake of osmoprotective substances. The expression level of the antioxidant enzyme systems is upregulated to scavenge reactive oxygen species. Simultaneously, the molecular chaperones *groES*/*groEL* and GB cooperate to maintain the functional stability of the protein. In this study, a trinity salt-tolerance-integrated strategy of “dynamic perception–hierarchical response–system synergy” of halophilic bacteria was initially proposed, which provided a research idea for exploring the salt–alkali-tolerant mechanism of halophilic bacteria and a theoretical basis for the further development and application of this strain.

## 1. Introduction

Soil salinization refers to the process by which soluble salts accumulate excessively in the soil from various sources [1]. The changes in physical and chemical properties caused by soil salinization can give rise to issues such as decreased soil fertility, reduced nutrient utilization rate, and poor air permeability, posing a considerable threat to the productivity and ecological sustainability of the soil ecosystem [2,3,4,5]. Currently, more than 800 million hectares of land globally are impacted by saline–alkali soil, while the total area of saline–alkali land in China is approximately 9.913 × 10^7^ hectares, accounting for about 10% of the global saline–alkali land area [6,7,8]. In recent years, the problem of soil salinization has been increasingly serious, making it particularly urgent to accelerate the improvement and utilization of saline–alkali land [9,10]. The existing physical and chemical improvement methods often entail problems such as large engineering quantities and high costs, whereas bioremediation technology boasts advantages like low cost, no secondary pollution, and high sustainability [11,12]. In biological improvement, the microbial remediation approach typically utilizes halophilic bacteria with the ability to adapt to adverse conditions to restore the ecosystem structure and function of saline–alkali soil [13].

The unique adaptability of halophilic bacteria in highly saline–alkali environments has made them the focus of research on the improvement of saline–alkali land [13]. Nevertheless, the current research on halophilic microorganisms mainly centers on extreme halophilic archaea, while there is relatively insufficient attention given to moderately halophilic bacteria, which have significant ecological significance [14]. Compared with extreme halophiles, moderately halophilic bacteria possess a broader range of salt concentration adaptation, higher metabolic functional diversity, and stronger application flexibility [15,16]. Their potential in the field of soil remediation is particularly prominent. Hence, an in-depth analysis of the salt-tolerance mechanisms of moderately halophilic bacteria holds significant theoretical and practical importance. Previous studies have indicated that moderately halophilic bacteria mainly adopt two osmotic adaptation strategies to cope with high-salt environments: one is the “ion balance” strategy, that is, maintaining the dynamic equilibrium of osmotic pressure inside and outside the cell by regulating the concentration of intracellular cations [17]; the other is the “synthesis and accumulation of compatible solutes” strategy, that is, actively accumulating compatible solutes such as betaine and trehalose in high-salt environments to prevent cell dehydration caused by high osmotic pressure and ensure the maintenance of their normal physiological functions [18,19,20]. Although previous studies have uncovered certain salt tolerance mechanisms of moderately halophilic bacteria—for instance, *Chromohalobacter salexigens* ANJ207 predominantly utilizes glycine betaine as the main osmolyte under high-salt stress, while *Halobacillis halophilus* accumulates chloride and compatible solutes to counter high-salt stress—currently, there remains a dearth of systematic and comprehensive analyses regarding the salt-tolerance mechanisms of moderately halophilic bacteria [17]. To further systematically disclose the molecular mechanisms of moderately halophilic bacteria in response to salt stress, this study conducted a comprehensive analysis of their gene expression patterns and metabolic regulatory networks by integrating genomic and transcriptomic sequencing data.

The research object in this study, *Oceanobacillus picturae*, was isolated and selected from soil samples of a desolate saline–alkali land in Dongying City. This strain is a typical moderately halophilic bacterium and has a wide distribution range. It has been discovered in multiple environments such as deep-sea algae, salt flats, shrimp paste, soy sauce, traditional fermented foods, human feces, and Arctic soil [21]. During the interaction between plants and microorganisms, it has been verified that *O. picturae* can promote the growth of red mangrove seedlings under drought stress and the growth of wheat seedlings under salt stress [21,22]. In previous research achievements, it was also disclosed that this strain possesses acid-producing characteristics, which hold significant practical application value for improving saline–alkali soil quality and promoting plant growth. However, the salt tolerance mechanism of this strain has not yet been clarified. This study mainly concentrates on aspects such as intracellular compatible solute molecules, salt-tolerant genes, and metabolic pathways, with the expectation of elucidating the salt-tolerant molecular genetic mechanism of this bacterium and its application potential. Utilizing the relevant osmotic adaptation mechanisms of *O. picturae* and the secondary metabolites it generates to develop new types of biological inoculants is expected to become one of the important innovative solutions for improving the problem of soil-alkali salinization in the near future [23].

## 2. Materials and Methods

### 2.1. Strain and Culture Conditions

Soil samples were collected from the saline–alkali land in Dongying, Yellow River Delta. The samples were homogenized with sterile water at a 1:10 (*w*/*v*) ratio, followed by centrifugation at 6000 rpm for 5 min to collect the supernatant. The supernatant was inoculated into LB liquid medium containing gradient-increasing NaCl concentrations (2% to 10% *w*/*v*). Halotolerant bacteria were screened through serial subculture with progressively elevated salinity. The enriched bacterial suspension from the highest NaCl concentration (10% *w*/*v*) was subjected to serial gradient dilution (10^−1^ to 10^−9^) and spread onto LB solid medium supplemented with 10% NaCl. After 48 h of incubation at 30 °C, individual colonies were purified via streaking plate method to obtain target halotolerant strains. To investigate the salt adaptation mechanisms of the strain, the purified isolates were inoculated into LB liquid medium supplemented with varying concentrations of NaCl (0% to 16% *w*/*v*) and cultured under controlled conditions of 30 °C and 150 rpm in an orbital shaker. OD_600_ values were measured at 5 h intervals over a 40 h period. Growth curves were plotted using kinetic data, and the salt stress response characteristics of the strains were systematically analyzed.

### 2.2. Phylogenetic Analyses

Genomic DNA was extracted by means of the Tianamp Bacteria DNA Kit (Tiangen Biochemical Technology (Beijing) Co., Ltd., Beijing, China), the 16S rRNA gene was amplified and subjected to sequencing. The resulting sequences were then subjected to BLAST (https://blast.ncbi.nlm.nih.gov/Blast.cgi) alignment within the NCBI GenBank database. The reference strain sequences exhibiting the highest homology were selected, and a phylogenetic tree was constructed via the neighbor-joining method using MEGA v11.0 software. Bacillus subtilis DSM 10 was designated as the outgroup, and the confidence of the nodes was validated through 1000 bootstrap replications.

### 2.3. Whole-Genome Sequencing, Assembly, and Annotation

Based on the PacBio Sequel II/PacBio Sequel IIe and Illumina NovaSeq PE150 platforms, single-molecule sequencing technology and the third-generation sequencer Nanopore were utilized for sequencing. Unicycler software (https://github.com/rrwick/Unicycler) was employed to combine the second-generation and third-generation data for assembly. Starting from the final assembly results (≥500 bp) of each sample, GeneMarkS software (version 4.17) (http://topaz.gatech.edu/) was adopted for coding gene prediction and filtering. The predicted genes were subjected to BLAST comparisons (blastp, evalue ≤ 1 × 10^−5^) against various functional databases. For the BLAST results of each sequence, the comparison result with the highest score (default identity ≥ 40%, coverage ≥ 40%) was selected for annotation. The commonly provided functional annotation databases mainly include GO (http://geneontology.org/), KEGG (http://www.genome.jp/kegg/), COG (http://www.ncbi.nlm.nih.gov/COG/), KOG, eggNOG, NR, TCDB, Swiss-Prot (http://www.ebi.ac.uk/uniprot/), CAZy, etc.

### 2.4. RNA Extraction, Library Preparation, and Sequencing

For transcriptomic analysis, strain DY09 was cultivated in LB medium containing 4% NaCl (L), 8% NaCl (C), 10% NaCl (M1), 12% NaCl (M2), and 20% NaCl (H) for 18 h. Subsequently, bacteria in the logarithmic growth phase were harvested by centrifugation using an ST16R centrifuge (Thermo Fisher Scientific, Waltham, MA, USA), and the total RNA of each sample was extracted using a kit (Tiangen Biotech Co., Ltd., Beijing, China). Then, the RNA samples underwent strict quality control, mainly using the Agilent 2100 bioanalyzer (Agilent Technologies, Palo Alto, CA, USA) to accurately detect the integrity and total amount of RNA. After the RNA passed the quality test, mRNA was enriched, and libraries were constructed in a strand-specific manner. After the library construction was completed, initial quantification was carried out using a Qubit 2.0 Fluorometer (Invitrogen, Carlsbad, CA, USA), and accurate quantification was performed using qRT-PCR. Once the libraries were qualified, Illumina sequencing was conducted.

### 2.5. RNA-Seq Data Analysis

Taking the whole-genome data of this strain as the reference genome, genomic location analysis of the filtered sequences was carried out using the Bowtie2 software (https://bowtie-bio.sourceforge.net/bowtie2/index.shtml). The HTSeq (v0.6.1) was employed to evaluate the gene expression levels. For samples with biological replicates, the DESeq2R software (1.20.0) was applied for differential expression level analysis, and the threshold for significantly differentially expressed genes was set as *p*-value < 0.05 and |log2FoldChange| > 1.0.

### 2.6. Validation of RNA-Seq Data by qPCR Analysis

To validate the RNA-seq data, quantitative real-time PCR (qPCR) analysis was conducted. Six genes were randomly selected to verify their expression levels under four different treatment conditions (4% NaCl, 8% NaCl, 12% NaCl, and 20% NaCl). Primers were designed using Primer Premier 5 software and synthesized by Shanghai Sangon Biotech Co., Ltd., Shanghai, China). The primer sequences of the target genes are presented in Supplementary Table S1. Total RNA was extracted using the RNAprep Pure Kit (Tiangen Biotech Co., Ltd., Beijing, China), and cDNA was reverse transcribed from the total RNA using the Hiscript III 1st Strand cDNA Synthesis Kit (+gDNA Wiper) Kit (Vazyme Biotech Co., Ltd., Nanjing, China). The q-PCR experiment was carried out on the LineGene 9600 PCR Detection System (Hangzhou Bioer Technology Co., Ltd., Hangzhou, China) under the following specific conditions: pre-denaturation at 95 °C for 5 min, 40 cycles including denaturation at 95 °C for 10 s, and annealing at 60 °C for 30 s. The internal reference gene was *rpoA*, and the relative gene expression levels were evaluated using the 2^−ΔΔCt^ method.

## 3. Result

### 3.1. Whole-Genome Sequencing Analysis of the DY09 Strain

**Figure 1:**
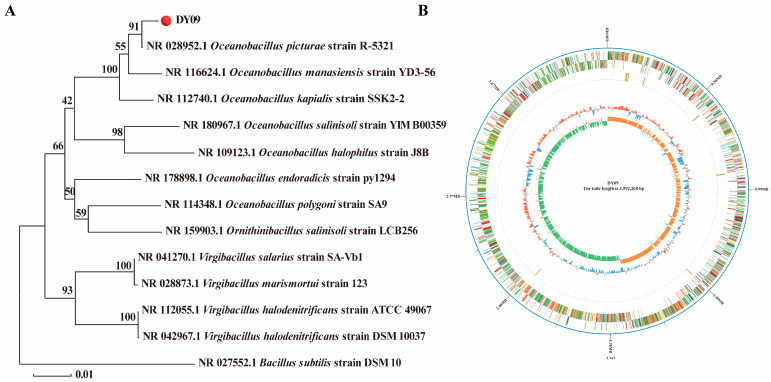
The results of whole-genome sequencing analysis of the DY09 strain. (**A**) Phylogenetic analysis of the DY09 strain. The phylogenetic tree was constructed based on the 16S rRNA gene sequence using the neighbor-joining method, and the reference sequences were downloaded from NCBI GenBank. (**B**) The whole-genome map of the DY09 strain.

Sequence alignment and phylogenetic analyses of the 16S rRNA sequence of strain DY09 were performed (Figure 1A). The findings revealed that strain DY09 falls within the genus Bacillus. It exhibits the closest genetic relatedness to strain R-5321 of *O. picture*, with a sequence similarity of 91%. The total amount of complete genome sequencing data was 2,686,349,705 bp, and the number of filtered reads was 347,068. The length of reads N50 was 10,206 bp, the length of reads N90 was 2882 bp, and the average read length was 7740 bp. Through assembly and annotation, a total of 4072 genes were obtained, with a GC content of 39.23%. The length of the complete genome was 3,992,268 bp, and a complete and gapless genome map was plotted (Figure 1B).

### 3.2. The Morphology and Growth Conditions of DY09 Strain

**Figure 2:**
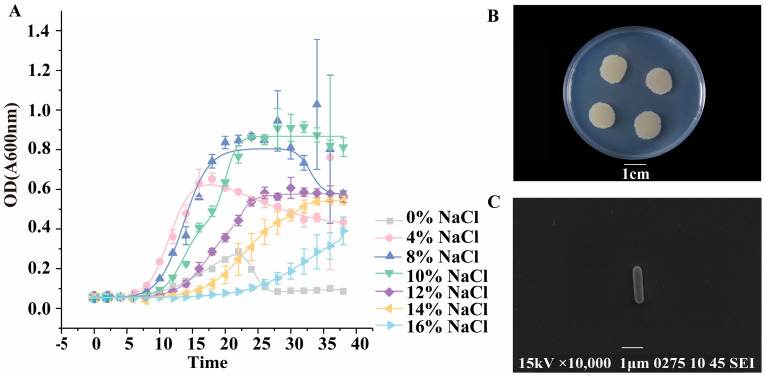
Morphology and growth conditions of the *O. picturae* DY09 strain from the grave painting. (**A**) Growth curves of the DY09 strain in media with different concentrations of NaCl, namely 0%, 4%, 8%, 10%, 12%, 14%, and 16% (*w*/*v*) NaCl. (**B**) Photographs of the colony morphology of the DY09 strain. Scale bar: 1 cm. (**C**) Scanning electron microscope photograph of DY09 strain. Scale bar: 1 μm.

To assess the salinity tolerance capacity of the DY09 strain, cells were cultivated within the NaCl concentration range of 0–16%, and the OD_600_ values were determined within 40 h to plot the growth curve (Figure 2A). The results demonstrated that the growth of the DY09 strain was inhibited when the salt concentration was either too low or too high. The suitable growth salt concentration range was 6–10%, and at 8% NaCl salt concentration, the biomass during the growth phase was significantly higher than that at other salt concentrations. Thus, the most suitable growth salt concentration for the DY09 strain was 8%, which classified it as a moderately halophilic bacterium. To identify the optimal carbon and nitrogen sources, 5 g/L of different carbon sources and 1 g/L of different nitrogen sources were added, respectively, at the optimal salt concentration. The results revealed that glucose and ammonium carbonate significantly promoted the growth of the DY09 strain and could be regarded as ideal carbon and nitrogen sources in the culture medium (Figure S1). Morphological observations indicated that the DY09 colony was milky-white, opaque, large, round, and had an irregular, wavy edge (Figure 2B). Scanning electron microscope images showed that the DY09 strain presented a long, rod-shaped structure with a smooth surface, typically with a length of 2.0–3.0 μm and a width of 0.2–0.5 μm (Figure 2C).

### 3.3. RNA-Seq Analysis of Transcriptome Expression of DY09 Strain Under Different Salt Stresses

**Figure 3:**
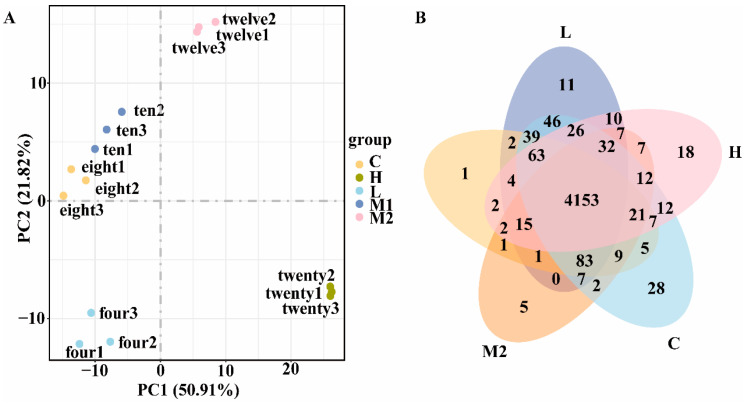
Results of gene quantitative analysis. (**A**) Venn diagram of co-expressed genes. (**B**) Two-dimensional clustering diagram of principal component analysis (PCA) among samples.

RNA-Seq analysis was performed under various salt concentrations (4, 8, 10, 12, and 20% NaCl, designated as Group L, Group C, Group M1, Group M2, and Group H, respectively) to explore the influence of salt stress on transcriptome expression. For each treatment condition, three biological replicate samples were acquired. The average number of raw reads obtained from each group was as follows: 17,803,248 bp for Group L, 15,875,651 bp for Group C, 16,666,105 bp for Group M1, 16,730,275 bp for Group M2, and 17,218,471 bp for Group H. After filtration, assessment of sequencing error rate, and examination of GC content distribution, the average number of clean reads obtained from each group was: 17,304,987 bp for Group L, 15,496,557 bp for Group C, 15,867,517 bp for Group M1, 15,842,555 bp for Group M2, and 16,614,318 bp for Group H (Table S2). As indicated in Supplementary Table S2, the base error rate of clean data for all samples was 0.03%, Q20 was higher than 97.3%, and Q30 was higher than 92.4%, signifying that the sequencing quality of the samples was high and appropriate for subsequent analyses. Subsequently, Fragments Per Kilobase per Million (FPKM)-mapped read values were utilized to evaluate the expression levels of genes in each sample, and the distribution of gene expression levels in different samples was presented through violin plots. The correlation coefficients within and between groups were calculated based on the FPKM values of genes in all samples and were depicted as a sample correlation heatmap (Figure S2). To further assess the differences between groups and the consistency of samples within groups, principal component analysis (PCA) was conducted on the gene expression values (FPKM) of all samples (Figure 3A). The results demonstrated that samples within the same group clustered together, suggesting that the variation within groups was minor and the repeatability was high, while Group L, Group M2, and Group H exhibited substantial differences between groups. In contrast, the samples in Group C and Group M1 presented a clustering tendency, indicating that the differences between these two groups were relatively small. Finally, the distribution of co-expressed genes in the five samples was displayed via a Venn diagram (Figure 3B), with a total of 4153 co-expressed genes. Additionally, in the samples of Group L, Group C, Group M1, Group M2, and Group H, there were 11, 28, 1, 5, and 18 genes that specifically responded to NaCl concentration, respectively.

### 3.4. Analysis of Differential Genes of DY09 Strain Under Different Salt Stresses

To deeply explore the transcriptome response of the DY09 strain under salt stress, three comparative schemes (Group L vs. Group C, Group M2 vs. Group C, and Group H vs. Group C) were designed in this study, with Group C serving as the control. We set the *p*-value < 0.05 and defined |log2FoldChange| > 1.0 as the threshold for significant differential gene expression (Table S3). In the comparison between Group L and Group C, a total of 719 differential genes were detected, among which 253 were upregulated and 466 were downregulated. In the comparison between Group M2 and Group C, 1652 differential genes were detected, with 821 being upregulated and 831 being downregulated. In the comparison between Group H and Group C, 2348 differential genes were detected, with 1150 being upregulated and 1198 being downregulated. Generally speaking, the number of differential genes increased correspondingly with the increase in salt concentration; however, regardless of whether it was under low-salt or high-salt stress conditions, the number of downregulated genes was always greater than that of upregulated genes. To visually represent the differential genes graphically, we plotted a volcano plot, which could clearly present the distribution of differential genes in each comparative combination (Figure S3).

**Figure 4:**
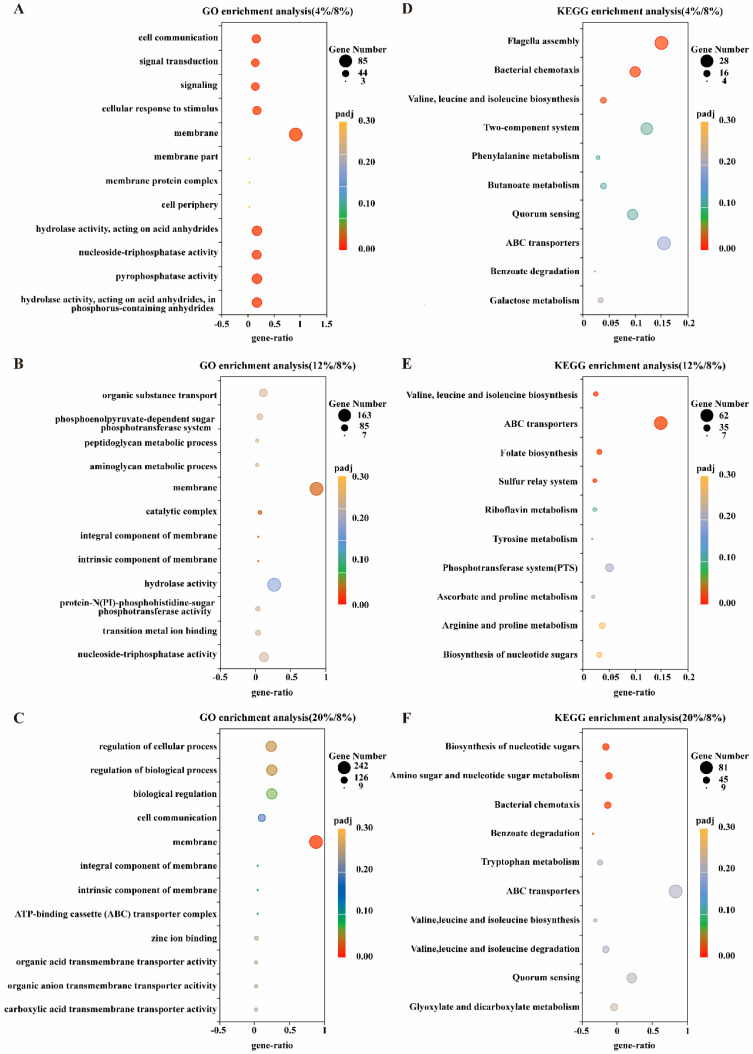
GO and KEGG enrichment analysis of the DEG set. (**A**) GO enrichment analysis of the DEG4%/8% set. (**B**) GO enrichment analysis of the DEG12%/8% set. (**C**) GO enrichment analysis of the DEG20%/8% set. (**D**). KEGG enrichment analysis of the DEG4%/8% set. (**E**) KEGG enrichment analysis of the DEG12%/8% set. (**F**) KEGG enrichment analysis of the DEG20%/8% set.

To identify the tolerance pathways of osmotic adaptation under diverse salinity stresses, we performed GO and KEGG enrichment analyses on three groups of samples (Figure 4). In the GO analysis, the L group/C group significantly enriched nine pathways, among which three pertained to the biological process category and six belonged to the molecular function category. The biological process category mainly encompassed signal transduction (GO:0023052, GO:0007165) and cell communication (GO:0007154), while the molecular function category was predominantly associated with hydrolase activity (GO:0016787), pyrophosphatase activity (GO:0016462), and ATPase activity (GO:0017111). The M2 group/C group did not significantly enrich any pathways; however, the bubble chart indicated that the differentially expressed genes were mainly concentrated in the plasma membrane (GO:0016020), catalytic activity (GO:1902494), plasma membrane protein complex (GO:0098797), transmembrane transporter complex (GO:1902495), and ABC transporter complex (GO:0043190). The H group/C group detected three significantly enriched pathways, of which two were annotated as biological processes and one as cellular component. The biological process category mainly involved regulation of cellular processes (GO:0050794) and regulation of biological processes (GO:0050789), while the cellular component category was correlated with the plasma membrane (GO:0016020). Generally speaking, the GO enrichment results reflected the alterations in gene expression related to signal transduction, ABC transporters, and the plasma membrane. In the KEGG analysis, the L group/C group significantly enriched three pathways, including flagellar assembly (BHA02040), bacterial chemotaxis (BHA02030), and branched-chain amino acid metabolism (BHA00290). As the salt concentration elevated, in the M2 group/C group, the pathways enriched by the differentially expressed genes mainly comprised sulfur relay system (BHA04122); folate biosynthesis (BHA00790); ABC transporters (BHA02010); valine, leucine, and isoleucine biosynthesis (BHA00290), arginine and proline metabolism (BHA00330); and oxidative phosphorylation (BHA00190). In the H group/C group, the specific pathways included biosynthesis of nucleoside sugar biosynthesis (BHA01250), amino sugar and nucleoside sugar metabolism (BHA00520), bacterial chemotaxis (BHA02030), and ABC transporters (BHA02010). In conclusion, the KEGG analysis results demonstrated that under different salt stress conditions, the gene expression in the pathways related to ABC transporters, cell chemotaxis, amino acid metabolism, and oxidative phosphorylation underwent significant changes.

### 3.5. The Role of Transmembrane Transport in Osmotic Adaptation

**Figure 5:**
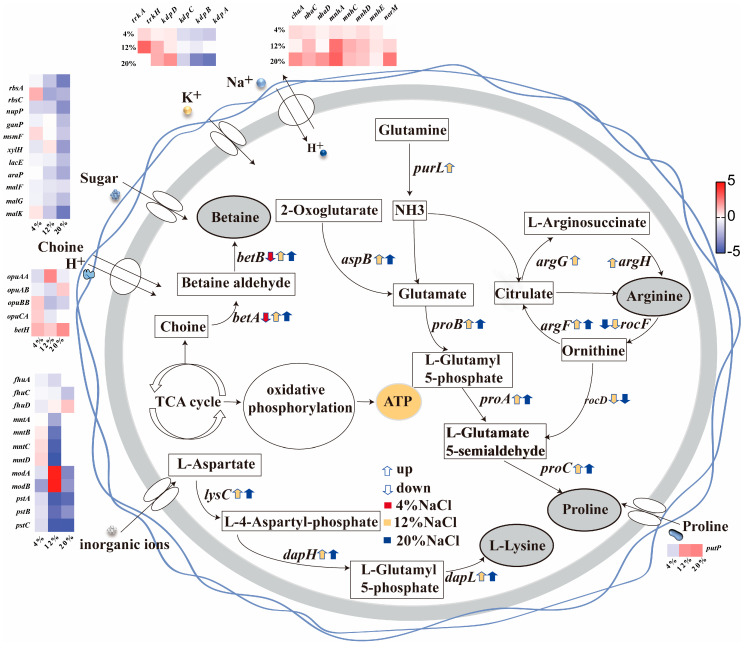
Schematic illustration of amino acid metabolism and membrane transport in strain DY09. For colored arrows, the direction indicates the upregulation or downregulation of gene expression, and the filled color reflects the expression under different salt concentrations, where red corresponds to 4% NaCl, yellow to 12% NaCl, and blue to 20% NaCl. The heat map reflects the differential expression profile (up-regulated genes are shown in red, down-regulated genes in blue, and no significant changes in white). For membrane transporters involved in salt-induced osmotic adaptation, please refer to Supplementary Table S4.

According to GO and KEGG enrichment analysis, we found that the DY09 strain possesses a substantial number of genes associated with transmembrane transport, indicating that transmembrane transport may play a crucial role in how halophiles respond to salt stress. The predominant strategy employed is the bacterial mechanism of “absorbing K^+^ while expelling Na^+^”. In the DY09 strain, two K^+^ influx systems were found: the constitutively expressed Trk system and the transcriptionally regulated Kdp system (Figure 5). The expression levels of genes encoding K^+^ uptake in both systems were not significantly different under 4% NaCl low-salt stress; however, under high-salt stress conditions of 12% and 20% NaCl, the expression levels of genes involved in K^+^ uptake within the Trk system (*trkA* and *trkH*) were significantly upregulated. Conversely, gene *kdpD* from the Kdp system was only upregulated under 20% NaCl high-salt stress, while no significant differences were observed for *kdpA*, *kdpB*, and *kdpC* across varying salt concentrations. This indicates that the DY09 strain primarily relies on the constitutive expression type Trk system for K^+^ uptake to cope with high salt stress. Sodium excretion predominantly depends on highly efficient Na^+^/H^+^ antiport proteins widely distributed among microbial systems that continuously extrude Na^+^, thereby maintaining stable intracellular sodium concentrations. In the DY09 strain, a series of genes related to Na^+^/H^+^ antiport proteins were found, including *chaA*, *nhaC*, *nhaD*, *nhaG*, *nhaP*, *mnhA*-*E,* and *norM*. The expression levels of genes related to Na^+^ excretion were basically unchanged under 4% NaCl low-salt stress compared with 8% NaCl; however, under 12% and 20% NaCl high-salt stress, the expression levels of genes encoding Na^+^/H^+^ antiport proteins (including *chaA*, *nhaC*, *mnhA*-*E*, and *norM*) were significantly upregulated. In summary, the elevated saline environment facilitated an increase in both sodium expulsion and potassium influx within cells as a joint response to heightened salinity stress; this strategy was not employed during periods of lower salinity.

Furthermore, the migration of mineral ions plays a crucial role in the response of halophiles to salt stress. Nevertheless, in the DY09 strain, salt stress seemingly has exerted a remarkable negative influence on the transport of iron, phosphate, manganese, and molybdate (Figure 5). Firstly, under salt stress conditions, iron is predominantly transported in the form of Fe^3+^, and the expression of the ABC transporter gene *fhuD* encoding the iron transport protein is significantly downregulated under 4% NaCl low-salt stress. The expressions of genes *fhuA* and *fhuC* encoding the iron transport proteins also demonstrate a distinct downregulation under 12% NaCl and 20% NaCl high-salt stress. The expressions of the ABC transporter genes *mntA*, *mntB*, *mntC*, and *mntD* encoding the phosphate transport proteins show no significant change under 4% NaCl low-salt stress but are significantly reduced to 4–7 times their original levels under 12% NaCl and 20% NaCl high-salt stress. The expressions of the ABC transporter genes *modA* and *modB* encoding the molybdate-related transport proteins remain stable under 4% NaCl low salt stress but fluctuate with the increase in NaCl concentration: they are upregulated under 12% NaCl salt stress but are significantly downregulated under 20% NaCl salt stress. Additionally, the expressions of the ATP-binding protein genes *pstA*, *pstB*, and *pstC* encoding the phosphate transport are significantly downregulated in different NaCl concentrations (including 4% NaCl, 12% NaCl, and 20% NaCl). Specifically, the expression is reduced by approximately one fold under 4% NaCl salt stress, while the reduction reaches about four folds under 12% and 20% high-salt stress. In general, whether under high salt stress or low salt stress, compared to the optimal 8% NaCl concentration, it will hinder the effective transport of mineral ions.

Apart from ion transport, the transportation of organic compounds is of equal significance for cells in responding to high-salt stress. As an energy source or compatible solutes, sugars are regarded as playing a crucial role in the cellular response to salt stress. Nevertheless, for the DY09 strain, the expression levels of ABC transporter genes related to sugars were relatively low under salt stress conditions (Figure 5). The illustration presents the alterations in the expression levels of ABC transporter genes associated with ribose (*rbsA* and *rbsC*), arabinogalactan/maltooligosaccharide (*ganP*), raffinose (*msmF*), lactose (*lacF*), xylose (*xylF*), arabinose (*araP*), and trehalose/maltose (*malK*, *malF*, and *malG*) under salt stress circumstances. The outcomes indicate that the expression level of the gene *rbsC* involved in ribose transportation is upregulated at 4% NaCl low-salt stress but is significantly downregulated when the salt concentration rises to 12% NaCl and 20% NaCl high-salt stress; the transport of xylose exhibits an opposite trend, with no obvious change in the expression level of the gene *xylF* at 4% NaCl low-salt stress but an upregulation at 12% NaCl and 20% NaCl high-salt stress. With the exception of xylose and ribose, the expression levels of ABC transporter genes for other sugars remain largely unchanged or demonstrate a downward tendency, suggesting that sugar transport does not notably influence the response of the DY09 strain to salt stress. However, it is noteworthy that under high salt stress conditions, the expression levels of transporter genes for some amino acids and their derivatives, such as the proline transporter gene *putP*, the glycine betaine transporter gene *betH*, the tetrahydropyrimidine transporter gene, etc., are significantly upregulated. These amino acids and their derivatives, as compatible solutes, may play a pivotal role in the survival of the DY09 strain in a high-salt environment.

### 3.6. The Role of Amino Acids in Osmotic Adaptation

**Figure 6:**
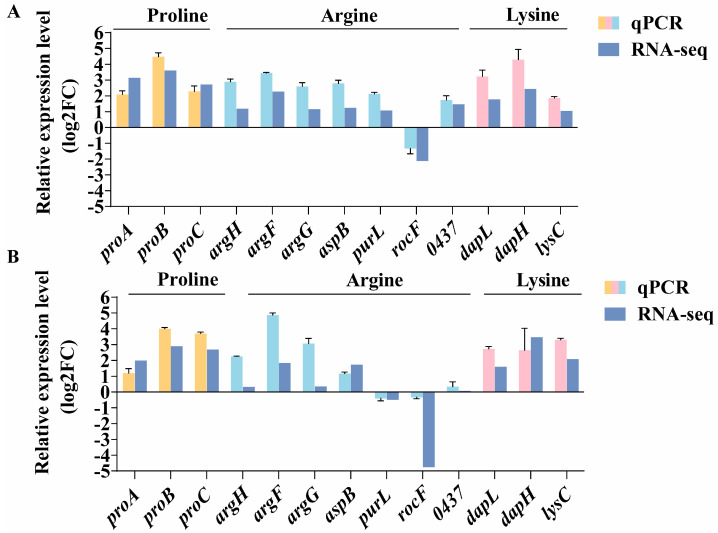
The role of amino acid metabolism in osmotic adaptation. (**A**) RNA-seq and qPCR results revealed the expression changes of genes related to proline, arginine, and lysine metabolism under 12% NaCl salt stress. (**B**) RNA-seq and qPCR results revealed the expression changes of genes related to proline, arginine, and lysine metabolism under 20% NaCl salt stress.

Amino acid-based organic osmoprotectants were among the earliest osmoprotectants identified in moderately halophilic bacteria, with common examples including proline, arginine, glutamic acid, and glutamine. Through KEGG enrichment analysis, we discovered that there were significant disparities in the amino acid metabolic pathways responded by the DY09 strain under varying salt stress conditions. In combination with transcriptome data (Table S5), we conducted a systematic analysis of the expression of genes related to amino acid metabolism in the DY09 strain under different salt concentrations, and the results are as follows: Under 4% NaCl low-salt stress, the biosynthesis of branched-chain amino acids (such as valine, leucine, and isoleucine) was inhibited, and the expression levels of the biosynthesis genes *ilvE* and *Novel00119* were significantly downregulated. Concurrently, the expression levels of the genes *Novel00120* and *GM003536* related to lysine degradation were significantly upregulated. This suggests that at lower salt concentrations, the bacteria might have reduced the demand for or synthetic capacity of branched-chain amino acids and regulated the internal metabolic balance by increasing the degradation of certain amino acids. Under 12% NaCl and 20% NaCl high-salt stress, it was observed that the expression of multiple genes involved in amino acid biosynthesis and degradation pathways underwent significant alterations. Specifically, the expression levels of the genes *aspB*, *argF*, *argG*, *argH*, *purl,* and *Novel00437* related to arginine biosynthesis were significantly upregulated, while the expression level of the arginine degradation-related gene *rocF* was significantly downregulated. Additionally, the expression levels of the genes *lysC*, *dapH,* and *dapL* related to lysine biosynthesis were significantly upregulated; the expression levels of the genes *proA*, *proB,* and *proC* encoding proline biosynthesis were also significantly upregulated. These outcomes were all verified by qPCR (Figure 6). Particularly under 20% NaCl high-salt stress, we further identified that the expression levels of the tryptophan biosynthesis gene *trpB* and the phenylalanine biosynthesis genes *pheA2* and *Novel00250* were significantly upregulated. The aforementioned changes indicate that at higher salt concentrations, bacteria enhance the synthesis of specific amino acids and reduce their degradation to facilitate their accumulation as compatible solutes within the cells, thereby effectively counteracting the influence of external high osmotic pressure.

### 3.7. The Role of Glycine Betaine in Osmotic Adaptation

**Figure 7:**
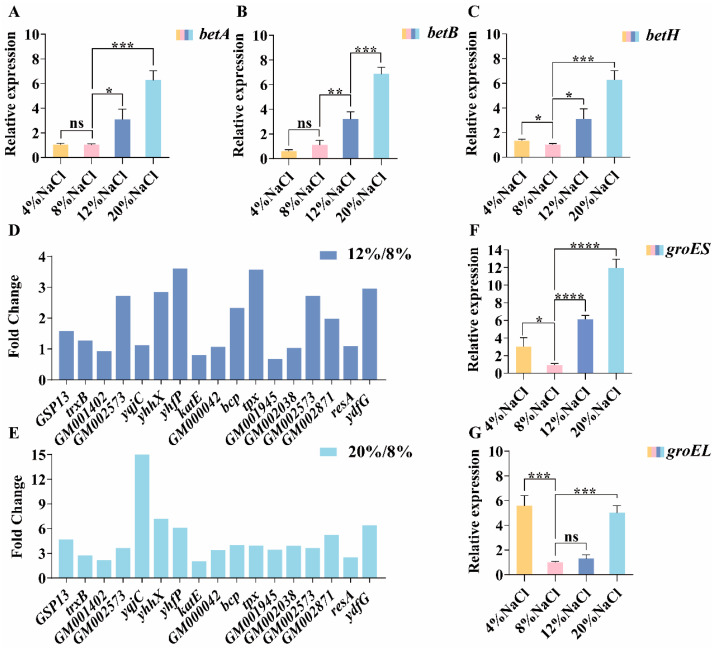
Differentially expressed genes (DEGs) involved in osmotic adaptation and oxidative stress. (**A**,**B**) qPCR of genes responsible for glycine betaine biosynthesis (*betA* and *betB*). (**C**) qPCR of genes representing those related to glycine betaine biotransport (*betH*). (**D**,**E**) Fold changes of differential genes representing those involved in oxidative stress conditions. (**F**,**G**) qPCR of heat-shock protein genes involved in heat-shock stress. In statistical significance annotation, the markers indicate: “ns”, *p* ≥ 0.05; “*”, 0.01 ≤ *p* < 0.05; “**”, 0.001 ≤ *p* < 0.01; “***”, 0.0001 ≤ *p* < 0.001; “****”, *p* < 0.0001.

Glycine betaine (GB), as an important compatible solute, plays a crucial role in the process of halophilic bacteria adapting to salt stress. In the DY09 strain, we discovered the genes involved in glycine betaine transport, namely *opuAA*, *opuAB*, *opuC*, *opuCA*, and *opuD*/*betH* (Figure 5). Analyzing the results of transcriptome sequencing (Table S6), it was found that the genes *opuAA*, *opuAB*, *opuC*, and *opuCA* involved in glycine betaine transport did not undergo significant changes. Therefore, we only quantified the *betH* gene for transporting glycine betaine (Figure 7C). Compared with the 8% NaCl concentration, under 4% NaCl salt stress, the expression level of the gene *opuD*/*betH* was upregulated by approximately 1.5 times; under 12% NaCl salt stress, it was upregulated by approximately 3 times; and under 20% NaCl salt stress, the expression level was even upregulated by approximately 6 times. The results indicate that the expression level of the glycine betaine transport gene *opuD*/*betH* shows an upward trend under both low and high-salt stress. Therefore, the glycine betaine transport gene *opuD*/*betH* plays an important role in the accumulation of GB as a compatible solute in the DY09 strain. Additionally, apart from uptake from the environment, betaine can also be accumulated through the oxidation of its precursor choline. We found that there are genes *betA* and *betB* that catalyze the conversion of choline to glycine betaine in the DY09 strain, suggesting that this strain largely relies on the choline oxidation pathway to synthesize glycine betaine. Therefore, we examined the expression of the two genes, *betA* and *betB*, by qPCR (Figure 7A,B). It was found that under 4% NaCl low-salt stress, the expression level of *betA* did not show significant changes, and the expression level of *betB* was significantly downregulated by approximately 2 times. However, under 12% NaCl and 20% NaCl high-salt stress, the expression levels of the synthetic genes *betA* and *betB* were both significantly upregulated several times. Combining the expression of the transport gene *opuD*/*betH*, under 4% NaCl low-salt stress, the expression level of the GB transport gene was upregulated, but the expression level of the synthetic gene did not change. Based on this, it is speculated that under low-salt stress, the DY09 strain may use GB as a carbon source and energy source. Under 12% NaCl and 20% NaCl high-salt stress, the expression levels of both the GB transport and synthetic genes were significantly upregulated. Thus, it is speculated that under high-salt stress, the DY09 strain will accumulate GB as a compatible solute to resist the high external osmotic pressure.

### 3.8. Other Differential Genes Participating in the Adaptation to Salt Stress

Salt stress can induce various stress responses in microorganisms. In addition to the aforementioned ionic stress and osmotic stress, a high-salt environment can also trigger oxidative stress. We have detected that under 12% and 20% NaCl high salt stress, the expression levels of genes associated with oxidative stress in the DY09 strain were significantly upregulated (Figure 7D,E). Particularly under 20% NaCl high salt stress, the fold increase in the expression of related genes was even more remarkable. For example, the expression level of the gene *GM002589* encoding a stress protein was upregulated by 2.23 times; the expression levels of genes related to detoxification mechanisms, such as *katE* encoding catalase, *tpx* encoding thiol peroxidase, *ydfG* encoding alkyl hydroperoxide reductase, and *GM000042* encoding superoxide dismutase, were all significantly elevated, ranging from approximately 2 to 6 times. Furthermore, the expression levels of genes related to the repair of oxidative damage, such as *yhhX* encoding a redox enzyme, *trxB* encoding thioredoxin reductase, genes encoding ferredoxin reductase, and *bcp* encoding a putative peroxiredoxin, also exhibited significant upregulation. Meanwhile, the expression level of the gene *yqjC* encoding the key enzyme 4-hydroxyphenylpyruvate dioxygenase for maintaining oxidative balance was upregulated by approximately 15 times. These results suggest that under high-salt stress conditions, the DY09 strain enhances its adaptability to the high-salt environment by upregulating the expression of a series of genes related to antioxidant defense and the repair of oxidative damage.

Salt stress not only elicits heat-shock stress, resulting in protein misfolding, but also activates the intracellular molecular chaperone protein system to assist in the correct folding of proteins and the formation of three-dimensional structures with biological activity. This multiple effect is of vital importance for the survival and functionality of cells. In the DY09 strain, the major heat-shock proteins we detected encompass Hsp33, Hsp60, Hsp70, Hsp90, etc. These heat-shock proteins play a crucial role in responding to environmental stress. Under salt stress conditions, the expression levels of the heat-shock protein genes *groES* and *groEL* in the Hsp60 family were significantly upregulated. Specifically, through qPCR quantitative analysis (Figure 7F,G), under 4% NaCl low-salt stress, the expression level of the groES gene was elevated approximately 6-fold, while that of the *groEL* gene was increased about 3-fold. This indicates that even under relatively low-salt stress, cells promptly activate emergency mechanisms to augment the expression of related genes, thereby enhancing their adaptability. Under 20% NaCl high-salt stress, the situation was even more remarkable: the expression level of the *groEL* gene was upregulated by 2-fold, and that of the *groES* gene was significantly upregulated by 12-fold. This substantial upregulation suggests that in extremely high salt environments, cells require more molecular chaperone proteins to assist in maintaining the normal folding of proteins, thereby protecting cells from the adverse impacts of high salt concentrations.

## 4. Discussion

Halophilic bacteria cope with salt stress by dynamically regulating intracellular ion homeostasis [24]. The core mechanism encompasses the synergy of the Na^+^/H^+^ antiporter system and the K^+^ uptake system [18,25,26,27,28]. This study discovered that the DY09 strain significantly upregulated the expression of single-subunit (*chaA*, *nhaC*, *nhaD*) and multi-subunit (*mnhA*-*E*) Na^+^/H^+^ antiporter proteins under high-salt stress (12%/20% NaCl), actively extruding Na^+^ to maintain a low intracellular sodium level. Such transport proteins are prevalently present in microorganisms, such as the chaA system in *E. coli* and the Mrp multi-subunit complex in *Pseudomonas aeruginosa* and *Bacillus subtilis* [29,30,31]. The encoding genes, *mnhA*-*E*, endow cells with resistance to Na^+^ and alkaline environments. Concurrently, the DY09 strain elevated intracellular K^+^ concentration by activating the K^+^ uptake system Trk (*trkA*, *trkH*) to balance osmotic pressure, while the ATP-dependent Kdp system demonstrated adaptive downregulation. This is in contrast to the regulatory pattern dominated by the KdpFABC system in the halophilic archaea *Halobacterium salinarum* and *Halobacterium* sp. NRC-1, suggesting that the Trk system is the main approach for high-salt adaptation in this strain [32]. Furthermore, the related genes of the Trk system were also identified in *Halomonas longissima* DSM 2581T [33]. It is notable that the genes of transport proteins for inorganic ions like iron and phosphorus are prevalently downregulated under high-salt stress. We hypothesize that the DY09 strain reduces the uptake of non-essential ions to lower the metabolic energy consumption. This selective regulatory strategy might be closely associated with its salt tolerance effect [18]. Moreover, studies have disclosed that the synergy of salt concentration and high pH would exacerbate cell damage, as the alkaline environment is liable to lead to the precipitation of metal ions, further suppressing the transport efficiency of mineral ions. This also accounts for the inhibition of the transport of inorganic ions such as iron and phosphorus by the DY09 strain in the saline–alkali soil environment [34].

Amino acid metabolism plays an important role in the adaptation of microorganisms to high-salt environments [18]. The DY09 strain copes with salt stress by dynamically modulating amino acid metabolism: under low-salt (4% NaCl) circumstances, it expedites lysine degradation and reduces the synthesis of branched-chain amino acids, potentially conserving energy by curtailing primary metabolism, whereas under high-salt (12% and 20% NaCl) stress, the synthesis genes of arginine, proline, etc, are significantly upregulated, and the degradation genes are suppressed, facilitating the accumulation of compatible solutes to counter osmotic pressure. It is notable that glutamate, as a common compatible solute, also plays a crucial role in certain microorganisms [35]. However, in the DY09 strain, the synthesis genes of glutamate (*gltB*/*D*/*S*) are generally downregulated. This is because glutamate can serve as a “second messenger” to stimulate the expression of proline genes and activate the proline synthesis pathway [36,37]. Specifically, under 20% NaCl conditions, the expression of the pyrroline-5-carboxylate reductase gene is upregulated by 4 times, driving the conversion of glutamate to proline [38]. This metabolic regulatory network discloses the dual roles of amino acids in high-salt adaptation: they not only directly act as osmoprotective substances but also achieve the dynamic balance of metabolic pathways through cascading regulation. The research findings suggest that the role of amino acids in the adaptation of microorganisms to high-salt environments is not merely limited to functioning as compatible solutes but also involves complex metabolic regulatory networks.

Glycine betaine (GB), serving as a core osmoprotectant for bacteria, is predominantly synthesized via two pathways: choline oxidation and glycine methylation [39,40]. Research indicates that the majority of halophilic bacteria retain merely one of these synthesis mechanisms. For instance, *Halomonas elongata* DSM3043 and *Halobacillus dabanensis* D-8T synthesize GB through the choline oxidation pathway: choline is transformed into betaine aldehyde by choline dehydrogenase encoded by *betA* and subsequently into GB by the dehydrogenase encoded by *betB* [41,42]. The genome of strain DY09 harbors the *betA*/*betB* gene cluster, validating that, similar to most Gram-positive bacteria, it adopts the choline oxidation pathway to predominantly orchestrate GB synthesis. At the ecological adaptation level, microorganisms have a propensity to acquire GB through transport systems to curtail energy consumption. For example, *Desulfovibrio* sp. prefers to transport GB rather than synthesize trehalose when GB is available in the environment. *Tetragenococcus halophilus*, lacking the key synthesis enzymes *gbsA*/*B*, wholly relies on exogenous GB uptake. The GB transport systems of Gram-positive bacteria mainly consist of the ABC superfamily (such as the *opuA*/*B*/*C* system) and the BCCT family (such as *betH*) [43,44]. Strain DY09 concurrently carries the opu system genes and the *betH* gene. However, analyses suggest that the BCCT transporter encoded by *betH* assumes a dominant role under high salt stress, which is in alignment with the osmotic adaptation strategies of strains like *Halobacillus trueperi*. Notably, the selection of synthesis and transport pathways by organisms holds significant ecological ramifications: when compatible solutes are present in the environment, relying on transport systems can promptly establish osmotic equilibrium, thereby attaining an advantage in interspecies competition, while retaining the synthesis pathway offers a survival guarantee in the face of extreme or solute-deficient circumstances. The coexistence of both mechanisms in strain DY09 might reflect its adaptive evolution to complex salinity environments.

It has been discovered that the DY09 strain adapts to salt stress through dynamic regulation of glycine betaine (GB) metabolism. In a high-salt environment (12–20% NaCl), the GB synthesis genes *betA*/*betB* are significantly upregulated, and concurrently, the transport gene *betH* is also upregulated, facilitating the accumulation of GB as a compatible solute to counteract osmotic pressure. Under low-salt conditions (4% NaCl), *betA*/*betB* are suppressed, but *betH* remains upregulated. At this point, the strain utilizes exogenous GB as a carbon and nitrogen source to sustain metabolism. This dual-mode regulation stems from the selective activation of metabolic pathways by osmotic pressure: high-salt inhibits catabolic enzymes and stimulates the activity of synthetic enzymes, while in a low-salt environment, catabolism takes the lead, prompting the strain to preferentially utilize GB for energy supply [45]. Notably, during high-salt stress, DY09 does not rely on the tetrahydropyrimidine system commonly found in Gram-positive bacteria; instead, the expression levels of its synthesis genes *ectA*/*B*/*C* decline. This might be associated with the competitive inhibition of GB precursors in the medium. The yeast medium preferentially initiates the GB synthesis pathway, thereby inhibiting the generation of other compatible solutes [40,46,47]. This phenomenon is in line with the typical osmoprotective strategy of moderately halophilic bacteria, suggesting that DY09 has evolved an osmotic adaptation mechanism centered on GB, achieving a functional transformation from low-salt metabolic support to high-salt osmotic protection through the coordinated regulation of synthesis and transport.

The challenges confronted by bacteria in high-salt environments encompass the damage to proteins, lipids, and nucleic acids inflicted by reactive oxygen species (ROS) [48,49]. To address the threat of ROS, the DY09 strain markedly upregulates the expression of catalase, peroxidase, and superoxide dismutase, reinforcing the antioxidant defense system and maintaining the redox balance, thereby enhancing the survival capacity of bacteria in extreme circumstances [50]. Molecular chaperone proteins play a crucial role in the response of bacteria to salt stress [51,52,53]. Members of the HSP60 family, *groES*/*groEL*, exhibit salt concentration-dependent upregulation (threefold in low salt and sixfold in high-salt) in the DY09 strain, while the expression of HSP70′s *dnaK* does not undergo a significant change, suggesting that HSP60 plays a predominant role in the salt adaptation of this strain. Furthermore, some of the literature points out that the accumulation of glycine betaine forms a synergetic mechanism with molecular chaperones: in the DY09 strain, glycine betaine accumulates continuously under high-salt stress. The expression level of *dnaK* is not significantly upregulated, but the expression levels of *groES* and *groEL* are significantly upregulated, collaborating with GB to inhibit protein aggregation and guarantee the normal function of cells [54,55]. This joint response mechanism of the antioxidant system, the molecular chaperone network, and compatible solutes discloses that bacteria maintain intracellular homeostasis to withstand high-salt stress through multiple strategies.

## 5. Conclusions

Overall, the salt tolerance mechanism of halophilic bacteria is manifested as an integrated strategy of “dynamic perception–hierarchical response–system synergy”. Specifically, the DY09 strain centers on the redistribution of metabolic resources under low salt stress, while under high salt stress, it rapidly attains osmotic balance through ion transport, combines amino acid metabolism and the synthesis and transport of GB, and takes into account both osmotic protection and the optimization of metabolic resources. Meanwhile, the antioxidant system, molecular chaperones, and compatible solutes form a “trinity” defense system, jointly safeguarding against oxidative damage, protein destabilization, and osmotic pressure imbalance, providing key survival guarantees for maintaining intracellular homeostasis of halophilic bacteria in salt stress environments. This study not only discloses the molecular basis of the cross-salinity adaptation strategy of the moderately halophilic DY09 strain but also holds multiple scientific values for guiding the development of microbial fertilizers for saline–alkali land, improving the salt tolerance of crops, and developing technologies for the biological remediation of saline–alkali land.

## Data Availability

The RNA-seq data reported in the current study are available in the NCBI SRA database (Bioproject: PRJNA1225427).

## Supplementary Materials

The following supporting information can be downloaded at https://www.mdpi.com/article/10.3390/microorganisms13071474/s1. Figure S1: Growth curves of DY09 strain under different carbon and nitrogen sources; Figure S2: Sample correlation analysis; Figure S3: Differentially expressed genes (DEGs) of *Oceanobacillus picturae* DY09 under salt stress; Table S1: Primers information of the genes for qPCR; Table S2: Statistics of quality control and sequence alignment results; Table S3: List of the number of transcripts upregulated or downregulated indifferent differential expression combinations obtained from the EdgeR program; Table S4: Membrane transport proteins participating in salt-induced osmotic adaptation; Table S5: Expression changes of Amino acid related genes under salt stress; Table S6: Expression changes of glycine betaine transport and synthesis genes under salt stress; Table S7: Expression changes of Oxidative stress and molecular chaperones genes under salt stress.
