# Supplementary material for: A Halophilic Bacterium for Bioremediation of Saline–Alkali Land: The Triadic and Synergetic Response Mechanism of Oceanobacillus picturae DY09 to Salt Stress
Source: Microorganisms. 2025 Jun 25;13(7):1474. doi: 10.3390/microorganisms13071474 (PMC12297972; doi:10.3390/microorganisms13071474)
Supplement: Supplementary file 1 [file microorganisms-13-01474-s001.zip › microorganisms-3681618-supplementary.pdf]

Supplementary Information

A Halophilic Bacterium for Bioremediation of Saline–Alkali Land: The Triadic and Synergetic Response Mechanism of *Oceanobacillus picturae* DY09 to Salt Stress

Tianying Nie, Liuqing Wang, Yilan Liu, Siqi Fu, Jiahui Wang, Kunpeng Cui and Lu Wang *

Institute of Biomedical Engineering, College of Life Sciences, Qingdao University, Qingdao 266071, China; ying18854709906@163.com (T.N.); 15751537912@163.com (L.W.); liuyilan2022@163.com (Y.L.);
fuussq@163.com (S.F.); jhwang@qdu.edu.cn (J.W.); cuikunpeng@qdu.edu.cn (K.C.)

* Correspondence: lwang@qdu.edu.cn

Table S1. Primers information of the genes for qPCR

Gene name	Primer sequence(5'-3')	Length
proA	F: CAGGCAGTCGTCAACAAT	300bp
	R: AGCCCAGTCATCTTCTCC	
proB	F: AAAATAATAGGAGTGGTGGAG	173bp
	R: AACAAGCAATACTTCGTGGC	
proC	F: TACAAACCGAAACAACCAAGA	202bp
	R: GATACCAGCCAATACAGAAATA	
argH	F: ATTTCAGCAGGTCTCAAGGT	193bp
	R: CACGTAAGTAGAGCCGCATA	
argF	F: TTACTGACCTTTATCATCCCTG	254bp
	R: CTCCACCACAATCTTTCCAC	
argG	F: CCAGCAGCCCTTACCTTG	252bp
	R: CCAGCAGCCCTTACCTTG	
aspB	F: ACATTATCACCCTCTTCCAC	213bp
	R: CTTATTCACAATCGCATCTT	
purL	F: CCTGGTTCCAACTGTGAT	149bp
	R: CGAAGGTAGTCTCCGTAT	
rocF	F: CCCGTTAGTATTCGGTGGTG	138bp
	R: GTTTCTGGCACCAATCTCCG	
Novel000437	F: GCAATGAAGCACCGACCTAT	212bp
	R: CCGAGCCAGAAAGCCACGATATTC	
dapL	F: AACCCGCCAGTAGATAAT	102bp
	R: GAACCACGCTTGGAACAC	
dapH	F: AAATTGGTGATGGCTGTG	119bp
	R: TTTACCTACTGTCGCTCTT	
lysC	F: ATGGTGGTAGGTGAAGGC	131bp
	R: ATCCCAAACATCATAGAAAC	
betA	F: GAAGGAACCCACGCAAAGG	254bp
	R: TGCTCCGAAACTGGCTCTG	
betB	F: GCCGTCCAAATGATCCAG	241bp
	R: ATACGCATCTTCGCCACA	
betH	F: TACCGAGCAGGCAATTAAGGA	155bp
	R: AAAGCGGAACAAGCGTAGCAC	
groES	F: GTTCTGGTCGCGTAA	114bp
	R: ATTCTGTGCCTTCATA	
groEL	F: AACCCTGAAGCAATCTC	247bp
	R: CAAGTGCAGTACCACCA	

rpoA	F: CGGTAGAAGTCAGCGAGGATG<br>R: AGTAGGATACGACGCAAGGAG	103bp

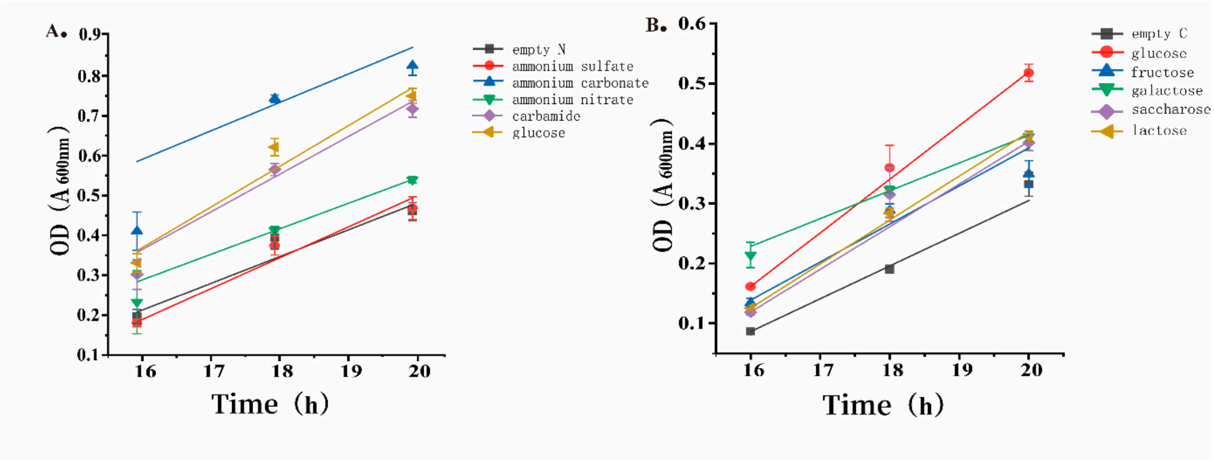

Fig. S1. Growth curves of DY09 strain under different carbon and nitrogen sources. A. Growth curves of DY09 strain under different nitrogen source conditions. The medium has an optimal salt concentration of 8%. B. Growth curves of DY09 strain under different carbon source conditions. The medium has an optimal salt concentration of 8%.

Table S2. Statistics of quality control and sequence alignment results.

Sample name	Raw reads	Clean reads	raw bases	clean bases	Error rate	Q20	Q30	GC content
eight1	15533524	15122906	2.34G	2.27G	0.03	97.66	93.15	40.47
eight2	16043540	15466460	2.41G	2.32G	0.03	97.76	93.25	40.81
eight3	16049890	15900306	2.41G	2.39G	0.03	97.58	92.82	40.37
twenty1	16183160	15694660	2.43G	2.36G	0.03	97.68	93.22	39.93
twenty2	18139008	17321974	2.73G	2.6G	0.03	97.51	92.87	40.39
twenty3	17333246	16826320	2.6G	2.53G	0.03	97.49	92.82	39.94
four1	16560262	15983148	2.49G	2.4G	0.03	97.36	92.47	40.66
four2	16166568	15796346	2.43G	2.37G	0.03	97.72	93.34	40.49
four3	20682914	20135468	3.11G	3.03G	0.03	97.58	93.05	41.35
ten1	18604736	17857894	2.8G	2.68G	0.03	97.4	92.47	40.82
ten2	12253414	11574844	1.84G	1.74G	0.03	97.69	93.05	40.37
ten3	19140164	18169814	2.88G	2.73G	0.03	97.8	93.38	40.39
twelve1	17666348	16787118	2.65G	2.52G	0.03	97.77	93.42	40.53
twelve2	17620122	17045130	2.65G	2.56G	0.03	97.74	93.18	40.26
twelve3	14904354	13695418	2.24G	2.06G	0.03	97.57	92.87	40.43

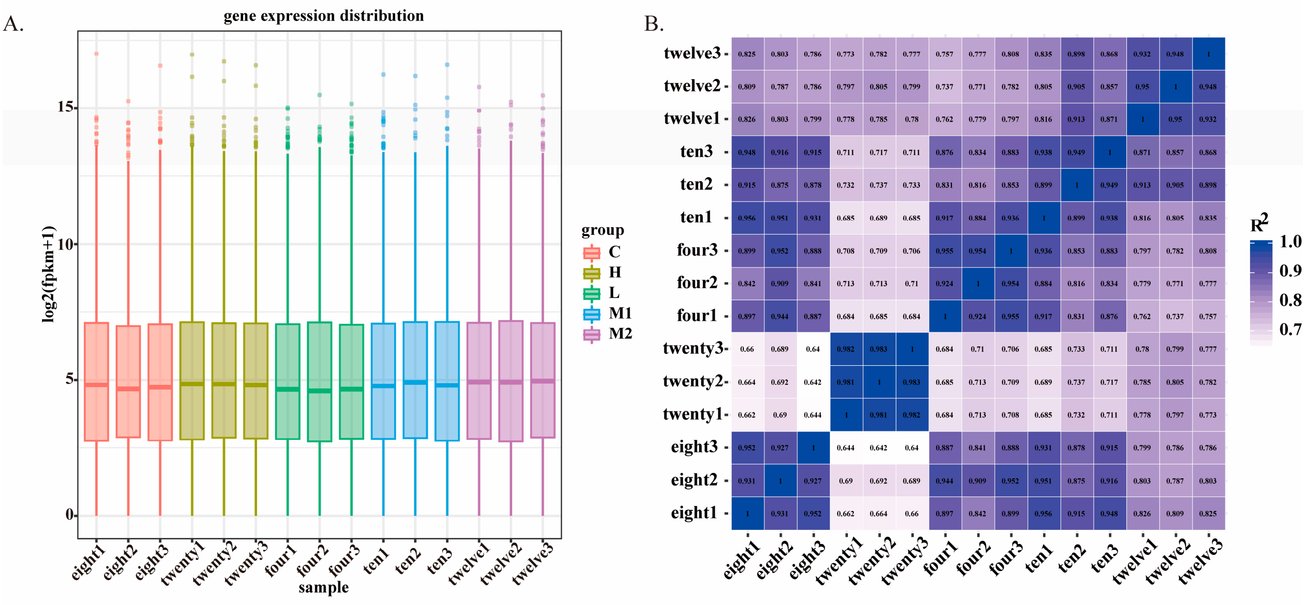

Fig. S2. Sample correlation analysis A. Distribution of gene expression levels in different samples B. Sample correlation coefficient correlation heat map

Table S3. List of the number of transcripts upregulated or downregulated indifferent differential expression combinations obtained from the EdgeR program.

Deseq Combination	Upregulated transcripts	Downregulated transcripts	Allregulated transcripts
HvsC	1150	1198	2348
M2vsC	821	831	1652
LvsC	253	466	719

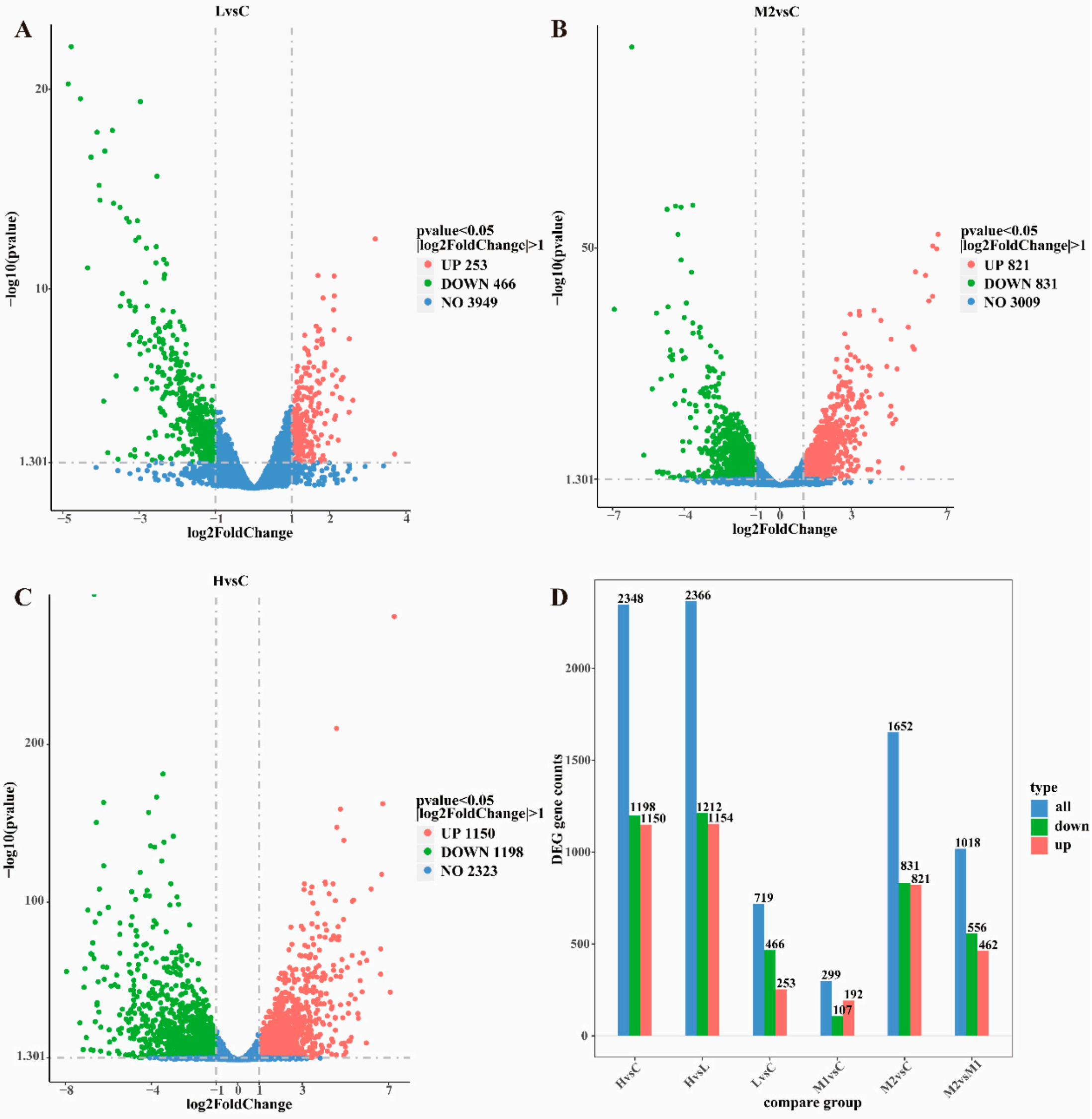

Fig. S3. Differentially expressed genes (DEGs) of *Oceanobacillus picturae* DY09 under salt stress. A-C Volcano plot of DEG sets. red: significantly up-regulated DEGs, green: significantly down-regulated DEGs, |log2FC| ⩾ 1, *p*-value < 0.05.D Differentially expressed genes for all comparison groups.

Table S4. Membrane transport proteins participating in salt-induced osmotic adaptation.

Gene id	Gene name	Gene description	Log_2(fold change)		
			4%/8%	12%/8%	20%/8%
GM002677	*chaA*	Na^+/H^+ antiporter	0.73	0.24	**2.15**
GM002216	-	Na^+/H^+ antiporter	-0.52	**1.27**	**3.33**
GM003197	*nhaC*	Na^+/H^+ antiporter	**-1.00**	**-1.62**	-0.96
GM001229	*nhaC*	Na^+/H^+ antiporter	-0.84	**1.33**	**1.50**
GM002622	*nhaC*	Na^+/H^+ antiporter	0.57	**1.27**	**1.55**
GM002622	*nhaC*	Na^+/H^+ antiporter	0.57	**1.27**	**1.55**
GM003894	*nhaG*	Na^+/H^+ antiporter	0.88	**-2.31**	**-1.06**
GM001229	*nhaC*	Na^+/H^+ antiporter	-0.84	**1.33**	**1.50**
GM001586	*nhaP*	Na^+/H^+ antiporter	0.21	**-1.73**	**-1.68**

GM002348	*nhaD*	Na^+/H^+ antiporter	0.24	0.13	**2.02**
GM003751	*nhaD*	Na^+/H^+ antiporter	-0.31	**1.34**	0.82
GM002290	*mnhA*	$Na(^+)/H(^+)$ antiporter subunit	0.57	**2.71**	**3.07**
GM002291	*mnhC*	$Na(^+)/H(^+)$ antiporter subunit	0.43	**1.49**	**1.25**
GM002292	*mnhD*	$Na(^+)/H(^+)$ antiporter subunit	0.66	**1.15**	**1.03**
GM002293	*mnhE*	$Na(^+)/H(^+)$ antiporter subunit	0.41	0.48	0.31
GM003610	*norM*	Na^+-driven multidrug efflux pump	-0.07	0.76	**2.56**
GM001640	*trkA*	Trk/Ktr K^+ transport system	-0.54	-0.32	**1.32**
GM001548	*trkA*	Trk/Ktr K^+ transport system	0.76	**3.02**	**6.17**
GM002629	*trkH*	Trk/Ktr K^+ transport system	0.26	**1.47**	**1.51**
GM001616	*trkH*	Trk/Ktr K^+ transport system	-0.38	**2.00**	**1.57**
GM000612	*kdpD*	K^+-sensing histidine kinase	**1.01**	0.87	**1.19**
GM001040	*kdpD*	K^+-sensing histidine kinase	0.67	**1.50**	-0.46
GM003182	*kdpD*	K^+-sensing histidine kinase	0.40	0.40	**2.13**
GM003974	*kdpC*	K^+-transporting ATPase	-0.95	-0.24	**-1.30**
GM003975	*kdpB*	K^+-transporting ATPase	**-1.23**	-0.57	**-3.51**
GM003976	*kdpA*	K^+-transporting ATPase	**-1.49**	-0.09	**-3.90**
GM003879	*fhuA*	ABC-type Fe^{3+} transport system	-0.39	**-1.11**	**-6.25**
GM003875	*fhuA*	ABC-type Fe^{3+} transport system	0.16	**-1.71**	**-4.76**
GM000463	*fhuA*	ABC-type Fe^{3+} transport system	0.49	**1.16**	-0.52
GM003733	*fhuB*	ABC-type Fe^{3+} transport system	-0.13	0.36	0.57
GM003759	*fhuC*	ABC-type Fe^{3+} transport system	-0.18	-0.34	**-1.70**
GM003731	*fhuD*	ABC-type Fe^{3+} transport system	-0.58	0.25	**1.08**
GM003734	*fhuG*	ABC-type Fe^{3+} transport system	-0.12	0.32	0.44
GM003767	*mntA*	manganese transport system	0.04	**-2.49**	**-6.69**
GM003768	*mntB*	manganese transport system	0.42	**-3.98**	**-6.98**
GM003769	*mntC*	manganese transport system	0.68	**-4.55**	**-7.12**
GM003770	*mntD*	manganese transport system	0.82	**-5.00**	**-7.16**
GM003513	*mntP*	manganese transport system	-0.20	0.67	0.84
GM002131	*mntR*	manganese transport system	-0.03	-0.35	-0.23
GM003939	*modA*	ABC-type molybdate transport system	-0.93	**4.65**	**-3.42**
GM004053	*modA*	ABC-type molybdate transport system	-0.88	-0.16	**-1.09**
GM003938	*modB*	ABC-type molybdate transport system	**-2.75**	**4.71**	**-3.06**
GM003701	*pstA*	ABC-type phosphate transport system	**-1.03**	**-4.62**	**-4.19**
GM003702	*pstB*	ABC-type phosphate transport system	-0.89	**-4.13**	**-3.17**
GM003700	*pstC*	ABC-type phosphate transport system	**-1.01**	**-4.74**	**-4.85**
GM003746	*rbsA*	Ribose import ATP-binding protein	-0.29	**-1.67**	**-3.69**
GM003745	*rbsC*	Ribose import ATP-binding protein	**1.50**	**-2.50**	**-2.13**
GM001854	*nupP*	Putative sugar ABC transporter, permease	**-1.22**	**-1.30**	**-3.23**
GM002846	-	sugar ABC transporter permease	0.75	0.99	**2.21**
GM002854	*ganP*	sugar ABC transporter permease	-0.37	0.05	**-1.89**
GM003048	-	ABC-type sugar transport system	**1.08**	0.31	**-2.59**
GM003104	*lplB*	ABC-type sugar transport system	-0.66	**-2.46**	**-1.47**
GM003130	*msmF*	ABC-type sugar transport system	0.69	-0.10	**-1.54**
GM003149	*msmF*	ABC-type sugar transport system	-0.70	**-1.49**	**-1.22**
GM003286	*gstB*	ABC-type sugar transport system	0.53	-1.00	-0.90

GM003320	*araP*	ABC-type sugar transport system	**-1.77**	**-1.45**	-0.69
GM003321	*araN*	ABC-type sugar transport system	-0.29	-0.42	**-1.82**
GM003336	*xylH*	Xylose transport system permease protein	0.14	**1.14**	**3.94**
GM003336	*xylH*	Xylose transport system permease protein	-0.71	0.47	-2.89
GM003286	*glcF*	Glucose transport system permease protein	0.53	**-1.00**	-0.90
GM003320	*lacF*	Lactose transport system permease protein	**-1.77**	**-1.45**	-0.69
GM003321	*lacE*	lactose/L-arabinose transport system	-0.29	-0.42	**-1.82**
GM003344	*araP*	ABC-type sugar transport system	0.03	**-1.24**	**-2.42**
GM003371	*malF*	Trehalose/maltose transport system	-0.37	-0.70	-0.89
GM003384	-	multiple sugar transport system substrate	0.49	0.16	-0.96
GM003385	-	Sugar abc transporter	**1.66**	-0.21	**-3.10**
GM002853	*malG*	ABC-type sugar transport system	-0.33	-0.93	**-1.86**
GM001237	*malK*	ABC-type sugar transport system	0.48	**-1.59**	**-3.80**

Bold, significant (|log2(fold change)| ≥ 1, p-value < 0.05)

Table S5. Expression changes of Amino acid related genes under salt stress.

Gene id	Gene name	Gene description	Log_2(fold change)		
			4%/8%	12%/8%	20%/8%
GM001133	*ilvE*	branched-chain amino acid aminotransferase	**-1.58**	0.45	**2.04**
Novel00119	-	branched-chain amino acid aminotransferase	**-1.16**	0.44	**2.27**
Novel00120	-	lysine degradation	**1.45**	-0.96	-0.39
GM003536	-	lysine degradation	**1.01**	0.64	-0.61
GM000428	*trpB*	tryptophan biosynthesis	0.15	-0.10	**1.13**
GM000977	*proA*	glutamate-5-semialdehyde dehydrogenase	-0.68	**3.13**	**1.98**
GM000978	*proB*	glutamate 5-kinase	-1.28	**3.61**	**2.90**
GM000979	*proC*	pyrroline-5-carboxylate reductase	-0.92	**2.72**	**2.68**
GM001980	*aspB*	aspartate aminotransferase	0.62	**1.23**	**1.73**
GM000688	*purL*	phosphoribosylformylglycinamidine synthase	-0.33	**1.07**	-0.49
GM000371	*argF*	Ornithine carbamoyltransferase	**-1.40**	**2.26**	**1.84**
GM003690	*argG*	argininosuccinate synthase	-0.46	**1.15**	0.35
GM003689	*argH*	argininosuccinate lyase	0.19	**1.18**	0.33
Novel00437	-	Nitric oxide synthase	-0.33	**1.47**	0.07
GM001652	*speA*	arginine decarboxylase	-0.92	0.81	-0.51
GM003540	*speB*	agmatinase	0.30	**-1.43**	**-1.20**
GM000230	*rocF*	arg arginase	-0.47	**-2.12**	**-4.75**
GM002101	*lysC*	aspartate kinase	-0.19	**1.04**	**2.08**
GM001634	*dapH*	tetrahydrodipicolinate N-acetyltransferase	0.26	**2.44**	**3.47**
GM001635	*dapL*	N-acetyldiaminopimelate deacetylase	0.36	**1.77**	**1.59**
GM002360	*pheA2*	prephenate dehydratase	0.54	0.32	**1.44**
Novel00250	-	prephenate dehydratase	-0.08	0.89	**1.57**
GM002360	-	prephenate dehydratase	0.54	0.32	**1.44**

Bold, significant (|log2(fold change)| ≥ 1, pvalue < 0.05)

Table S6. Expression changes of glycine betaine transport and synthesis genes under salt stress.

Gene id	Gene name	Gene description	Log_2(fold change)		
			4%/8%	12%/8%	20%/8%
GM001254	*opuAA*	glycine betaine transport ATP-binding protein	**-1.00**	**2.27**	-0.49
GM000970	*opuAB*	glycine betaine transport ATP-binding protein	-0.44	**-1.02**	0.97
GM001908	*opuBB*	glycine betaine transport ATP-binding protein	0.98	**-1.86**	**-1.30**
GM001907	*opuCA*	glycine betaine transport ATP-binding protein	0.91	-0.67	-0.05
GM000406	*betH*	glycine betaine transporter	**1.43**	0.96	**2.13**
GM003133	*betL*	glycine betaine transporter	0.90	**1.56**	**2.68**

Bold, significant (|log2(fold change)| ≥ 1, p-value < 0.05)

Table S7. Expression changes of Oxidative stress and molecular chaperones genes under salt stress.

Gene id	Gene name	Gene description	Log_2(fold change)		
			4%/8%	12%/8%	20%/8%
GM002589	*GSP13*	general stress protein	0.86	0.66	**2.23**
GM002030	*trxB*	thioredoxin reductase	**1.05**	0.35	**1.46**
GM001402	-	thioredoxin reductase	0.57	-0.12	**1.1**
GM002573	-	thioredoxin reductase	-0.05	**1.44**	**1.86**
GM002088	*yqjC*	4-hydroxyphenylpyruvate dioxygenase	**1.73**	0.16	**3.9**
GM003908	*yhhX*	oxidoreductase	-0.91	**1.51**	**2.85**
GM003303	*yhfP*	Putative quinone oxidoreductase	0.85	**1.85**	**2.6**
GM000334	*katE*	catalase	0.56	**-1.16**	**1.23**
GM000574	*katE*	catalase	0.64	**-1.12**	**1.64**
GM001340	*katE*	catalase	0.47	-0.32	**1.02**
GM000042	-	superoxide dismutase activity	0.57	0.09	**1.76**
GM000923	*bcp*	Putative peroxiredoxin	0.39	**1.22**	**1.99**
GM001898	*resA*	Thiol-disulfide oxidoreductase	0.07	0.12	**1.31**
GM002508	*tpx*	thiol peroxidase	-0.43	**1.83**	**1.97**
GM003133	*ydfG*	Alkylhydroperoxidase family enzyme	0.9	**1.56**	**2.68**
GM001945	-	ferredoxin oxidoreductase	-0.28	-0.56	**1.78**
GM002038	-	Ferredoxin	0.8	0.04	**1.96**
GM002573	-	Ferredoxin	-0.05	**1.44**	**1.86**
GM002871	-	ferredoxin oxidoreductase	0.88	0.98	**2.39**
GM000591	*groES*	Molecular chaperone (HSP60)	**1.69**	**1.36**	**2.65**
GM000592	*groEL*	Molecular chaperone (HSP60)	**1.36**	0.67	**1.56**
GM002267	*danK*	Molecular chaperone (HSP70)	0.58	-0.21	0.78

Bold, significant (|log2(fold change)| ≥ 1, p-value < 0.05)
